# Effects of Transcranial Direct Current Stimulation (tDCS) on Cognitive Performance and Cerebral Oxygen Hemodynamics: A Systematic Review

**DOI:** 10.3389/fnhum.2021.623315

**Published:** 2021-04-07

**Authors:** Mathieu Figeys, Michael Zeeman, Esther Sung Kim

**Affiliations:** ^1^Faculty of Rehabilitation Medicine, University of Alberta, Edmonton, AB, Canada; ^2^Faculty of Medicine and Dentistry, University of Alberta, Edmonton, AB, Canada; ^3^Department of Communication Sciences and Disorders, University of Alberta, Edmonton, AB, Canada

**Keywords:** cognition, transcranial direct current stimulation (tDCS), functional near infrared spectroscopy (fNIRS), aging, cerebral perfusion, cerebral hemodynamic functional response

## Abstract

**Background:** There is increasing evidence to support the efficacy of transcranial direct current stimulation (tDCS) applications in cognitive augmentation and rehabilitation. Neuromodulation achieved with tDCS may further regulate regional cerebral perfusion affiliated through the neurovascular unit; however, components of cerebral perfusion decrease across aging. A novel neuroimaging approach, functional near-infrared spectroscopy (fNIRS), can aid in quantifying these regional perfusional changes. To date, the interaction of the effects of tDCS on cognitive performance across the lifespan and obtained fNIRS hemodynamic responses remain unknown.

**Objective:** This review aims to examine the effects of tDCS on cognitive performance and fNIRS hemodynamic responses within the context of cognitive aging.

**Methods:** Six databases were searched for studies. Quality appraisal and data extraction were conducted by two independent reviewers. Meta-analysis was carried out to determine overall and subgroup effect sizes.

**Results:** Eight studies met inclusion criteria. The overall effect size demonstrates that tDCS can alter cognitive performance and fNIRS signals, with aging being a potential intermediary in tDCS efficacy.

**Conclusion:** From the studies included, the effects of tDCS on cognitive performance and fNIRS metrics are most prominent in young healthy adults and appear to become less robust with increasing age. Given the small number of studies included in this review further investigation is recommended.

## Introduction

Interventions to enhance cognitive functioning are increasingly being used as a potential avenue to combat the effects of dementia and age-related cognitive decline. These range from behavioral training programs to non-invasive brain stimulation (Butler et al., 2018; Song et al., 2018; Zhang et al., 2019; Chou et al., 2020). Transcranial direct current stimulation (tDCS), one type of non-invasive brain stimulation, involves the application of a low-dose electrical current across the brain. tDCS is often paired with behavioral training protocols and is hypothesized to alter the efficacy of training-induced cognitive performance. Increasing evidence suggests that tDCS acts beyond neuronal structures and may modulate cerebral perfusion (Stagg et al., 2013). The relationships between the mechanisms of cognition, cerebral perfusion, and neuronal activity remain poorly understood, especially when considering healthy and pathological cognitive aging. With the use of functional near-infrared spectroscopy (fNIRS) to measure key factors in perfusion, as well as cognitive performance metrics, the impact of aging on these mechanisms can be explored. The purpose of this systematic review is to begin to explore the effects of tDCS on cognitive performance and fNIRS signals, with an emphasis on how these may differ across age.

### Non-invasive Electrical Brain Stimulation

Among available transcranial electrical current stimulation modalities, tDCS, and transcranial alternating current stimulation (tACS) are the most commonly reported techniques within the literature (Polanía et al., 2018). Direct current (DC) stimulation is utilized in tDCS, compared to an oscillating sinusoidal-current at a set frequency used in tACS. The physiological effects of tACS neuromodulation are thought to target specific neuronal frequency bands (Polanía et al., 2018), compared to neural polarity modulation involving voltage-dependent ion channels in tDCS (Nitsche et al., 2003). These differences in electrical properties may result in different neurophysiological responses. In this review, we focus on the cognitive and cerebral perfusion effects of tDCS, in combined tDCS and fNIRS protocols.

### Transcranial Direct Current Stimulation

Transcranial direct current stimulation (tDCS) is one form of non-invasive brain stimulation that has been used in numerous healthy and clinical populations (Meinzer et al., 2015; Prehn and Flöel, 2015; Smith et al., 2015; Cappon et al., 2016; Berryhill and Martin, 2018; Ke et al., 2019; Martinotti et al., 2019; Matar et al., 2020). Low-dose direct current applied to the brain is thought to modulate resting membrane threshold with application-dependent stimulation montages producing a differential increase or decrease in neuronal excitability (Nitsche and Paulus, 2000, 2001; Paulus, 2011; Stagg and Nitsche, 2011). The effects of tDCS are often examined using behavioral task metrics but reported results have been variable (Prehn and Flöel, 2015; Cappon et al., 2016; Woods et al., 2018; Rose et al., 2020).

Neuronal modulation induced by tDCS works in a summative fashion across neurons. Anodal tDCS is believed to invoke hypopolarization without reaching the depolarization threshold, whereas cathodal stimulation is thought to further shift the neuron into a hyperpolarized state (Stagg and Nitsche, 2011). These effects have proven beneficial in cognitive studies across aging and clinical populations; anodal tDCS has been demonstrated to increase performance on working memory (Ohn et al., 2008), cognitive control (Boudewyn et al., 2020), and language (Flöel et al., 2008). In contrast, cathodal stimulation has been demonstrated to decrease cognitive control (Wolkenstein et al., 2014). Thus, the potential clinical utility of tDCS targeting cognitive augmentation in aging and in cognitive disorders such as Mild Cognitive Impairment (MCI) may be of significant value.

tDCS can be easily paired with other treatment modalities, including cognitive rehabilitation protocols. For instance, researchers have reported that anodal-tDCS paired with cognitive training in young adults resulted in higher performance on a working memory task compared to the sham condition (Ke et al., 2019). Although these findings are promising, wide variability in terms of results and effect sizes exists within the tDCS literature. Numerous methodological variables including tDCS dosage, location, and length of stimulation, as well as population parameters such as age, education, and health status, may impact reported results. Overall, a consensus seems to be emerging that there is no clear advantage of adding tDCS to cognitive protocols (Cruz Gonzalez et al., 2018). Even with this uncertainty, the use of tDCS has been demonstrated to increase regional blood flow in those receiving tDCS paired with cognitive training (Das et al., 2019). Therefore, tDCS may potentially evoke other physiological and neurological mechanisms beyond behavioral responses.

Working memory is a cognitive function, which has been shown to be affected by age-related changes (Dickstein et al., 2013). In turn, aging may impact the efficacy of tDCS during working memory tasks. In a meta-analysis specifically examining the effects of tDCS on working memory in healthy young adults, no significant differences in performance were reported (Mancuso et al., 2016). However, when tDCS was paired with cognitive training, a small yet significant effect size was observed on working memory performance (Mancuso et al., 2016). A separate study investigating the effects of tDCS on working memory in older adults reported increased functional connectivity in the group receiving active anodal stimulation compared to the sham stimulation group during an *n-*back task (Nissim et al., 2019). Despite the increase in functional connectivity in the anodal group, no significant differences in performance were noted on the *n*-back task (Nissim et al., 2019).

Age and disease status may play a pivotal role in tDCS outcomes, including aging-related cognitive disorders. A meta-analysis conducted by Hsu et al. (2015) examined the effects of non-invasive brain stimulation, including tDCS, on cognitive function in healthy older adults and those with Alzheimer's dementia. A small effect size was reported in healthy older adults, and a large effect size was found in older adults with Alzheimer's (Hsu et al., 2015). Similar results in healthy older adults were reported by Summers et al. (2016) with a moderate effect size. When examining effect sizes obtained across studies, there appears to be a trend of tDCS augmenting performance to a greater degree in those with lower cognitive functioning. That is, older adults with cognitive impairment seem to receive a greater benefit than healthy older adults, who in turn receive a greater benefit than young healthy adults (Hsu et al., 2015; Mancuso et al., 2016; Summers et al., 2016; Nissim et al., 2019). This finding should be interpreted with caution, however, as methodological and population variability is present across studies included within the published literature.

### Cerebrovascular Perfusion Changes Across Aging and tDCS Considerations

In addition to neuro-cognitive modulation, tDCS may invoke cerebroperfusional modulation associated with cortical hemodynamic functions (Zheng et al., 2011; Takai et al., 2016; Quinn et al., 2020). However, the interaction between tDCS induced effects on cognition and cerebral perfusion across aging remains widely unknown. Post-tDCS cerebral perfusion changes have been measured using neuroimaging techniques such as functional magnetic resonance imaging (fMRI) (Antal et al., 2011) and functional near-infrared spectroscopy (fNIRS) (Patel et al., 2020). Widespread decreases in cerebral perfusion after cathodal and anodal tDCS have been reported using arterial spin labeling (Stagg et al., 2013). Furthermore, regional decreases in blood-oxygen-level-dependent signals have been reported beyond, but not within, the region of stimulation (Antal et al., 2011). Regarding fNIRS, significant interindividual and methodological variability on reported tDCS effects exists in tDCS-fNIRS study designs (Patel et al., 2020). However, increases in cortical activation are reported during resting state; interestingly, a decreased level of cortical activation has also been reported during online tasks (Patel et al., 2020).

Changes in cerebral blood flow and cerebrovascular structure such as plaque formation, rarefaction, and vascular-wall connectivity appear to be aging dependent [see Sonntag et al. (2019) for an overview]. Moreover, disorders impacting both systemic and cerebral vasculature are associated with pathological age-related cognitive decline (Gasecki et al., 2013; Hardigan et al., 2016; Iadecola and Gottesman, 2019). Current evidence suggests a decrease in cerebral blood flow occurs in individuals with MCI beyond the extent of normal cognitive aging (De Eulate et al., 2017; Leeuwis et al., 2018; McKetton et al., 2019; Kim et al., 2020), yet it remains unclear whether this is an accompanying or a causal factor. Consequently, normal and pathological vascular changes may impact tDCS-evoked neuromodulation and cerebral perfusion modulation in older adults relative to young adults. Ultimately, when considering the potential effects of tDCS on cognitive performance and cerebral perfusion, different responses may occur across age and disease status.

It is important to consider structures and mechanisms beyond the neuron and their potential impacts on cognition, such as the neurovascular unit. The neurovascular unit comprises a dynamic interaction between the neuron, vasculature, and glial cells (Iadecola, 2004); the mechanism in which tDCS directly acts upon the neurovascular unit beyond the neuron itself remains unclear. Applied stimulation appears to alter vessel diameter to accommodate for the regional increase in neuronal metabolism (Iadecola et al., 1997). tDCS may also alter astrocytic mediated responses resulting in downstream vascular responses (LeMaistre Stobart et al., 2013). tDCS induced perfusional modulation occurs across cortical and subcortical structures (Stagg et al., 2013). Thus, perfusion changes may underlie behavioral-induced tDCS effects (Stagg et al., 2013), potentially through neurovascular coupling.

Investigating the interaction of cerebral perfusion and cognition, total cerebral blood flow appears to decrease across healthy aging. In an investigation of cerebral perfusion and cognitive aging, Catchlove et al. (2018) report a cerebral blood flow difference of roughly 84.15 mL min^−1^ between the younger and older adult groups. Interestingly, the investigators reported an interaction between total cerebral blood flow and attention in older adults, but not in younger adults. This interaction between cognitive performance and cerebral blood flow in older adults demonstrates an unexpected inverse relationship, with increased performance associated with a decrease in cerebral blood flow, potentially suggesting higher neural efficiency mechanisms (Catchlove et al., 2018).

There appears to be a trend toward declining cerebral blood flow in older adults with pathological cognitive impairment. Kitagawa et al. (2009) report a statistically significant lower cerebral blood volume in older adults with cognitive impairment compared to cognitively healthy age-matched controls. In addition to certain subcortical structures, significant differences in frontal, temporal, parietal, and occipital cortices were all present between groups differing in cognitive status (Kitagawa et al., 2009). Similarly, significantly lower cerebral blood flow was reported in older adults with Alzheimer's dementia compared to those with subjective cognitive impairment (Leijenaar et al., 2017).

Again, a general trend may be arising from the literature, suggesting that the greatest tDCS modulation of cerebral blood flow occurs in healthy young adults, followed by healthy older adults, and finally older adults with cognitive impairment. Note, this is in the opposite direction of the previously hypothesized trend of tDCS impacting behavioral performance to a greater degree in those with cognitive impairments. To summarize, the neurophysiological mechanisms of tDCS may act downstream on the neurovascular unit. When tDCS is applied, both neuronal and perfusional modulation occurs. As vasodilation results in a localized influx of blood, these perfusional changes may be quantified using fNIRS.

### Functional Near-Infrared Spectroscopy

fNIRS is a novel functional neuroimaging technique that utilizes near-infrared light to measure hemoglobin chromophores (oxyhemoglobin; HbO, deoxyhemoglobin; HbR, and total hemoglobin; HbT) (Wilcox and Biondi, 2015). Concentrations of each chromophore can be calculated by applying the measured optical properties in a modified Beer-Lambert equation (Wilcox and Biondi, 2015). Under normal circumstances, cortical activation increases oxyhemoglobin concentration with an associated decrease in deoxyhemoglobin concentration (Wilcox and Biondi, 2015). These concentrations can quantify local perfusion changes within the first few centimeters of the brain cortex and has been previously correlated with fMRI BOLD signals (Huppert et al., 2006). fNIRS has been used increasingly within cognitive neuroscience research, and signal responses are sensitive to both cognitive load and cognitive state (Fishburn et al., 2014). As fNIRS primarily measures the superficial cerebral structures composed of gray matter (Quaresima et al., 2005; Bigio and Fantini, 2016) it can be a useful neuroimaging tool for examining the effects of tDCS.

fNIRS has several advantages over other neuroimaging methods. fNIRS devices tend to be more cost-efficient than an fMRI or EEG, user-friendly, and increasingly portable (with lightweight wireless options that can pair over Bluetooth). fNIRS is advantageous in that it can control for movement and be applied to individuals who have contraindications for MRI (Obrig, 2014; Almajidy et al., 2020), and may be better tolerated by older adults (Stephens and Berryhill, 2016). While the temporal resolution is significantly higher than fMRI, spatial resolution is limited to the superficial layers of the cortex (Obrig, 2014; Almajidy et al., 2020). Given this expanding area of research, further discussion regarding the utility of fNIRS in cognitive paradigms as a function of aging is required.

### Purpose

Previous studies have successfully utilized fMRI with tDCS during cognitive tasks, though only a handful have implemented fNIRS with tDCS [see Patel et al. (2020) for a review]. As methodological and perfusional considerations differ between fNIRS protocols and other types of neuroimaging, this study will solely review tDCS-fNIRS protocols targeting cognition. Specifically, the purpose of this systematic review is to explore the neuromodulatory effects of tDCS delivery on cognitive performance and oxygen hemodynamics. Furthermore, the variable of age will be explored across reported metrics. The proposed research questions are as follows:

1. Does tDCS alter cognitive performance and regional oxygenation during cognitive tasks as measured by fNIRS?
2. Does aging impact the efficacy of tDCS on cognitive performance and fNIRS signals?

Based on the literature, it is hypothesized that tDCS effects on cognitive performance will be greater in older adults compared to younger adults. Regarding fNIRS metrics, we hypothesize young adults will experience greater perfusional change than older adults due to decreasing cerebral blood flow rates in aging.

## Methods

### Search Strategy

Electronic searches were conducted using the following databases: CINAHL, Embase, Medline, PsychInfo, Pubmed, Scopus, and Web of Science using Boolean operators in consultation with a research librarian. Search terms included (transcranial direct current stimulation OR tDCS) AND (near-infrared spectroscopy OR functional near-infrared spectroscopy OR fNIRS). This search method resulted in all available tDCS and fNIRS articles; cognitive-orientated studies were then manually extracted. Database searches were conducted on February 19, 2020, and updated on December 27, 2020. No date restrictions were placed on the literature search. Compiled results were imported into Covidence (Covidence Systematic Review Software, Veritas Health Innovation, Melbourne, Australia), where inclusion and exclusion criteria were applied.

### Inclusion and Exclusion Criteria

Full-text journal articles published in English were included if they applied tDCS (either concurrent or sequential) and fNIRS to a cognitive paradigm. Non-cognitive study protocols (such as motor function) and review articles were excluded. Further, articles were included if they reported baseline and post-tDCS stimulation metrics on both cognitive performance and recorded fNIRS signals. To compare the efficacy of tDCS, studies were included if they reported a control (sham) and treatment group, or a crossover design study. No restrictions were placed on tDCS type, duration, current intensity, or time of stimulation. Other non-invasive brain stimulation methods such as transcranial magnetic stimulation and transcranial alternating current stimulation were excluded as physiological effects may differ from tDCS. Within this review focusing on cognition, articles reporting healthy adults, or older adults with MCI or dementia were included, with no boundaries on age limits. All other medical diagnoses and mental health disorders were excluded. If studies reported additional metrics in addition to a cognitive paradigm, only the reported interaction between tDCS on performance and fNIRS recordings within the context of the cognitive domain was included within the analysis.

### Quality Assessment

Each article was reviewed and underwent quality appraisal by two independent reviewers. Six articles were found in the initial search, and two additional articles were included in the updated literature search. Appraisal checklists were selected according to study design using The Joanna Briggs Institute Critical Appraisal Checklist for Randomized Controlled Trials (Joanna Briggs Institute, 2017) or the Ding et al. (2015) checklist for crossover design. Traditional quality appraisal tools may bias crossover research designs, hence to minimize bias, the proposed checklist outlined in Ding et al. (2015) was applied. Quality assessment tools for other study designs were not required for the final selection of articles due to a relative homogeneity in study designs. Discrepancies in the quality assessment were discussed and resolved. Scores were assigned to each study according to checklist criteria to allow for comparison. Fleiss's kappa was calculated in SPSS Version 26 (IBM Corporation, Armonk, NY, USA) to determine the initial inter-reliability between the reviewers.

### Meta-Analysis

Appropriate statistical values for effect size calculations (including: means, medians, standard deviations, standard errors, *p*-values, *F*-Values, and regression coefficients) in addition to sample sizes were extracted from the identified articles. Data was extrapolated from reported figures when necessary. Cohen's *d* effect sizes were calculated for the changes in cognitive performance and fNIRS signals reported within each study. If regression-based beta-estimates were reported without an r value, an estimated r value was calculated using the criteria outlined by Peterson and Brown (Peterson and Brown, 2005). This imputed r value was then utilized within the conventional effect size analysis outlined by Cohen (Cohen, 1988). Effect sizes were interpreted as: small (*d* = 0.2), medium (*d* = 0.5), and large (*d* = 0.8).

These effect sizes were then imported into Stata (Version 16; StataCorp, College Station, TX, USA) to further process and run the meta-analysis. To investigate the variable of age, a subgroup meta-analysis was performed. A random-effects model using restricted maximum likelihood was utilized to conduct the meta-analysis. REML minimizes bias while reducing mean squared error compared to other meta-analysis approaches (Langan et al., 2019). It should be noted that with the small number of studies present with varying protocols, a high level of heterogeneity is suspected. We will report overall heterogeneity *I*^2^ statistics, however, REML derived point-heterogeneity in limited meta-analysis sample sizes should be interpreted with caution and reported with confidence intervals (Von Hippel, 2015; Langan et al., 2019).

## Results

### Study Selection

**Figure 1:**
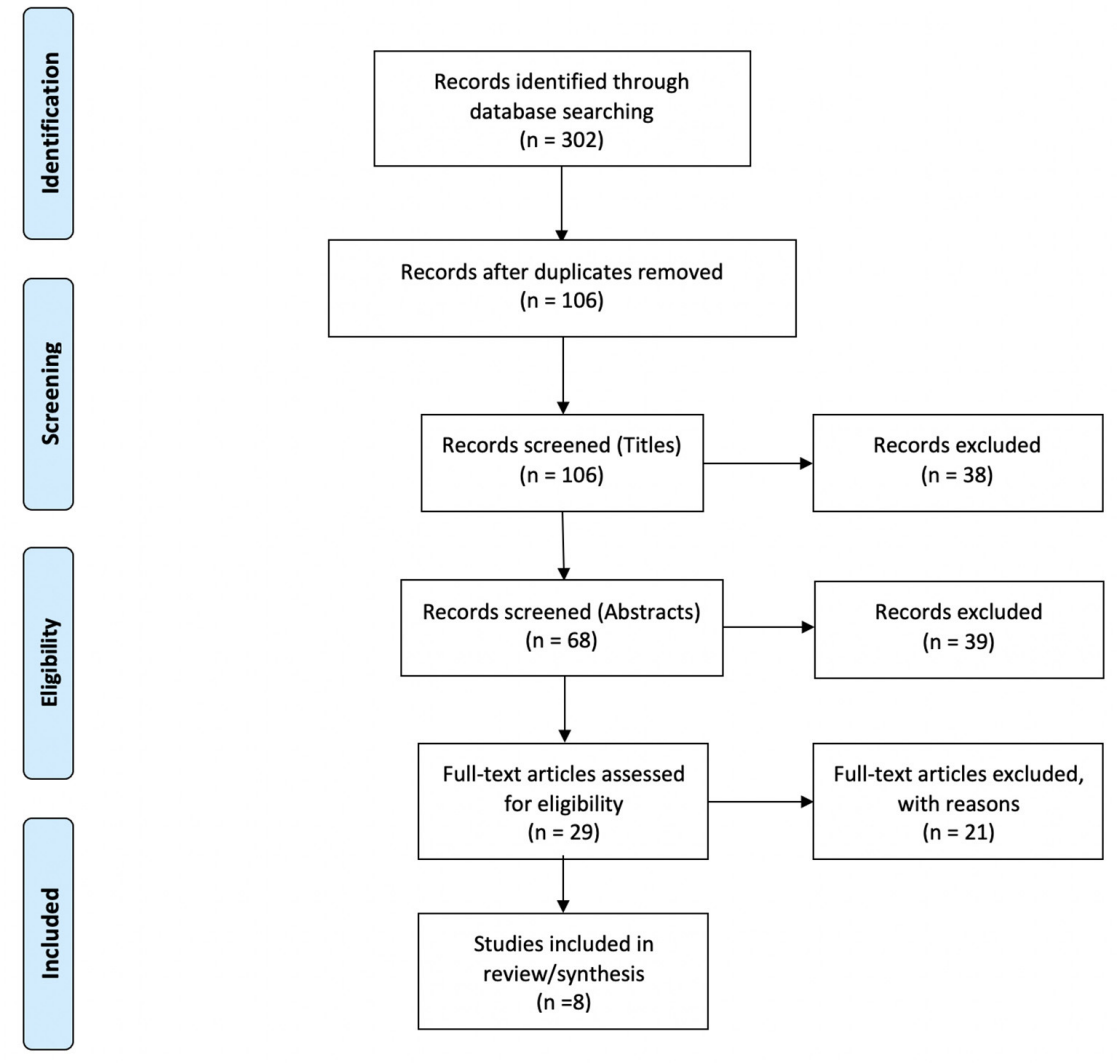
PRISMA flow diagram.

Of the 302 references identified during the initial database search, 196 duplicates were removed. 106 studies were screened, 29 of which underwent full-text review. Twenty-one articles were excluded for the following reasons: lacking a cognitive protocol (*n* = 9), wrong patient population of interest (*n* = 4), not an empirical research study (*n* = 4), lacking a fNIRS protocol (*n* = 2), lacking application of tDCS (*n* = 1), and lacking cognitive task measures with fNIRS (*n* = 1) resulting in eight studies suitable to be included within the review (Jones et al., 2015; Choe et al., 2016; Ehlis et al., 2016; Stephens and Berryhill, 2016; Herrmann et al., 2017; Borragán et al., 2018; Di Rosa et al., 2019; McKendrick et al., 2020). Please refer to the PRISMA diagram in Figure 1 for details. Table 1 describes the participant demographics across all included studies.

**Table 1:** Characteristics of participants.

**Reference**	**Population**	**Exclusion criteria**	**Sex**	**Mean age (SD if reported)**	**Mean education (SD)**
Borragán et al., 2018	Healthy young adults (*n* = 22; 20 in final sample)	Poor sleep quality; moderate-high usual CF (cognitive fatigue), excessive sleepiness, excessive anxiety/depression	8M, 14F	23 (2.28)	NR
Di Rosa et al., 2019	Healthy older adults (60–80 years) (*n* = 24; 21 in final sample) (*Experiment 1)	History of neurological/psychiatric illness, contraindications to tDCS, left-handed	9M, 12F	69.7 (5.1)	14.1 (3.3) years
Ehlis et al., 2016	Healthy young adults; (Group 1 *n* = 23; Group 2 *n* = 23)	Left-handed, history of mental/neurologic disorders, contraindications to tDCS	1: 9M, 14F 2: 12M, 11F	1: 32.1 (10.5) 2: 24.3 (2.4)	NR
Jones et al., 2015	Healthy young adults (*n* = 24) (*Experiment 1)	Neurological/psychiatric symptoms or head injuries; medications	12M, 12F	23.8 (3.7)	NR; University students
Herrmann et al., 2017	Healthy young adults (*n* = 61)	Mental, neurological, or psychiatric illness; current use of psychopharmaceuticals, contraindications to tDCS	31M, 30F	24.3	NR; 55 College students; 6 with 10 years of education
Stephens and Berryhill, 2016	Healthy older adults (*n* = 90; 30 in each group Sham, Active1–1 mA, Active2−2 mA)	Neurologic/psychiatric diseases, contraindications to tDCS, seizure disorders, medications, MMSE <22	Sham: 14M, 16F	Sham: 69.9	Sham: 15.2 years
			Active1: 14M, 16F	Active1: 68.6	Active1: 15.8 years
			Active2: 13M, 17F	Active2: 68.6	Active2: 15.7 years
Choe et al., 2016	Healthy adults (*n* = 32) DLPFC Active: *n* = 7 DLPFC Sham: *n* = 7 M1 Active: *n* = 10 M1 Sham: *n* = 11	Poor visual acuity, history of epipetic seizures, history of known neurological disorders, pregnancy (or likely to become pregnant during the study)	M: 31 F: 1	DLPFC Stim: 35 (11) DLPFC Sham: 42 (13) M1Stim: 41 (16) M1Sham: 31 (5)	NR
McKendrick et al., 2020	Cognitively healthy young adults (Sham: *n* = 10; Active: *n* = 11)	Current use of psychopharmaceutical agents	M: 10	20.3	NR; University students
			F: 11		

*NR, Not Reported; M, Male; F, Female; MMSE, Mini-Mental State Examination; M, Male; F, Female*.

### Quality Assessment

Quality scores ranged widely depending on the appraisal tool used. Four articles were appraised using the Ding et al. (2015) crossover study checklist, and each had a total score of 3/9, though the scoring of individual items varied (see Table 2) (Jones et al., 2015; Ehlis et al., 2016; Borragán et al., 2018; Di Rosa et al., 2019). Four articles were appraised using the JBI Critical Appraisal Checklist for Randomized Controlled Trials (Joanna Briggs Institute, 2017) with a mean score of 10/13 (Choe et al., 2016; Stephens and Berryhill, 2016; Herrmann et al., 2017; McKendrick et al., 2020). The mean quality percent score of all articles was 57.5% with a range of 33.3–84.6%. Descriptions of the individual items and corresponding scores are described in Tables 2, 3. Inter-rater reliability was considered strong with a Fleiss' κ of 0.851.

**Table 2:** Quality assessment—crossover studies.

**References**	**Checklist from Ding et al. (****2015****) for cross-over studies**									**Total score**
	**(1)**	**(2)**	**(3)**	**(4)**	**(5)**	**(6)**	**(7)**	**(8)**	**(9)**	
Borragán et al., 2018	1	−1	0	0	0	1	1	1	0	3/9
Di Rosa et al., 2019	1	−1	0	0	0	1	1	1	0	3/9
Ehlis et al., 2016	1	0	0	0	1	1	0	0	0	3/9
Jones et al., 2015	1	−1	0	0	0	1	1	1	0	3/9
Total item score	4/4	−3/4	0/4	0/4	1/4	4/4	3/4	3/4	0/4	

*Each item was scored according to the risk of bias: 1 low risk, 0 unclear, −1 high risk*.

*(1), Appropriate crossover design; (2), Randomized treatment order; (3), Carryover effect; (4), Unbiased data; (5), Allocation concealment; (6), Blinding; (7), Incomplete outcome data; (8), Selective outcome reporting; (9), Other bias*.

**Table 3:** Quality assessment—randomized control trials.

**Reference**	**JBI critical appraisal checklist for randomized controlled trials (Joanna Briggs Institute**, **2017****)**													**Total score**
	**(1)**	**(2)**	**(3)**	**(4)**	**(5)**	**(6)**	**(7)**	**(8)**	**(9)**	**(10)**	**(11)**	**(12)**	**(13)**	
Herrmann et al., 2017	1	0	0	1	1	1	1	1	1	1	1	1	1	11/13
Stephens and Berryhill, 2016	1	0	1	1	0	0	1	1	1	1	1	1	1	10/13
Choe et al., 2016	1	0	0	1	1	0	1	1	1	1	1	1	1	10/13
McKendrick et al., 2020	1	0	0	1	0	0	1	1	1	1	1	1	1	9/13
Total item score	4/4	0/4	1/4	4/4	2/4	1/4	4/4	4/4	4/4	4/4	4/4	4/4	4/4	

*Each item was scored according to answer: 1 yes, 0 unclear or N/A, −1 no*.

(1), Was true randomization used for assignment of participants to treatment groups? (2), Was allocation to treatment groups concealed? (3), Were treatment groups similar at baseline? (4), Were participants blind to treatment assignment? (5), Were those delivering treatment blind to treatment assignment? (6), Were outcomes assessors blind to treatment assignment? (7), Were treatment groups treated identically other than the intervention of interest? (8), Was follow up complete and if not, were differences between groups in terms of their follow up adequately described and analyzed? (9), Were participants analyzed in the groups to which they were randomized? (10), Were outcomes measured in the same way for treatment groups? (11), Were outcomes measured in a reliable way? (12), Was appropriate statistical analysis used? (13), Was the trial design appropriate, and any deviations from the standard RCT design (individual randomization, parallel groups) accounted for in the conduct and analysis of the trial?

### Impact of tDCS on Cognitive Task Outcomes

All eight studies reviewed investigated anodal tDCS compared to sham stimulation, with two of these studies also including a cathodal tDCS stimulation condition (Ehlis et al., 2016; Herrmann et al., 2017). Only two articles reported an increase in immediate cognitive performance (Di Rosa et al., 2019; McKendrick et al., 2020). A third study reported no increase in cognitive performance, however, an increase in an untrained task at 1-month follow-up was evident, dependent on dose (i.e., the greatest increase in those receiving 2 mA, followed by 1 mA, compared to sham) (Stephens and Berryhill, 2016). There were no reported effects of tDCS on verbal fluency task performance. The two studies which included older adult participants (Stephens and Berryhill, 2016; Di Rosa et al., 2019) both reported improvements in cognitive performance. Only one of the six studies with young adult participants reported an increase in accuracy and precision on a spatial memory task (McKendrick et al., 2020). tDCS parameters and cognitive effects are presented in Table 4.

**Table 4:** tDCS parameters and effects on cognition.

**Reference**	**Montage**	**Participant grouping**	**Age (SD)**	**# tDCS sessions**	**Active tDCS parameters**	**Region stimulated**	**tDCS administration (Online/offline to cognitive task)**	**Significant changes in cognitive performance?**
Borragán et al., 2018	Anodal/Sham	Within Subject	23 (2.28)	1 Active/1 Sham	1.5 mA for 25 min	Anode: left dorsolateral prefrontal cortex (F3) cathode: right forearm	Online	No
Di Rosa et al., 2019	Anodal/Sham	Within Subject	69.7 (5.1)	1 Active/1 Sham	1.5 mA for 26 min	Left PFC between F3 and F7; reference on contralateral shoulder	Online	Yes: Anodal tDCS with reward motivation increased WM performance (Baseline WM as a modulator)
Ehlis et al., 2016	Anodal/Sham, Cathodal/Sham	Within Subject	(1) 32.1 (10.5) (2) 24.3 (2.4)	1 Active/1 Sham	1 mA for 20 min	Broca's area (between C3, F3, F7); reference on contralateral supraorbital region	Offline to VFT	No
Jones et al., 2015	Anodal/Sham	Within Subject	23.8 (3.7)	1 Active/1 Sham	1.5 mA for 10 min	Anode over left prefrontal cortex (between F3 and F7); cathode over the contralateral cheek	Offline	No
Herrmann et al., 2017	Anodal, Sham, Cathodal	Between Group	24.3 (NR)	1	1.5 mA for 26 min	Bilateral Prefrontal Cortex	Online	No
Stephens and Berryhill, 2016	Anodal/Sham	Between Group	Sham: 69.9 (NR) Active2: 68.6 (NR) Active2: 68.6 (NR)	5	1 or 2 mA (two separate groups) for 15 min	Anode over F4; reference on the contralateral cheek	Offline (tDCS was paired with WM training)	*n*-back, No significant differences, however, a trend was seen in the Active2 group of increased benefit *2 mA tDCS did significantly increase far transfer tasks after 1 month
Choe et al., 2016	Anodal/Sham	Between Group	DLPFC Stim: 35 (11) DLPFC Sham: 42 (13) M1Stim: 41 (16) M1Sham: 31 (5)	4	2 mA for 60 min	Right dorsolateral prefrontal cortex: Anodes: F6 and FC6; Cathodes: Fp2, AF4, and AF8 Left Motor Cortex: Anodes: CP1 and CP3; Cathodes: Fp1, F9, F8	Online (motor finger-tapping task done prior)	*n-*Back, No significant differences between DLPFC stimulation condition as well as M1 stimulation conditions on accuracy *Reduced variability within individual learning rates with DLPFC stimulation, however, the trend appears to be minimal with M1 stimulation.
McKendrick et al., 2020	Anodal/Sham	Within Subject and Between Group	20.3 (NR)	2 Control: Sham & Sham Active: Sham & Active	1 mA for 15 min	Right ventrolateral prefrontal cortex: Anode over F10; cathode over F2	Online	Yes: Anodal tDCS increased spatial memory task performance

*NR, Not Reported; WM, Working Memory; VFT, Verbal Fluency Task; DLPFC, Dorsolateral prefrontal cortex*.

**Figure 2:**
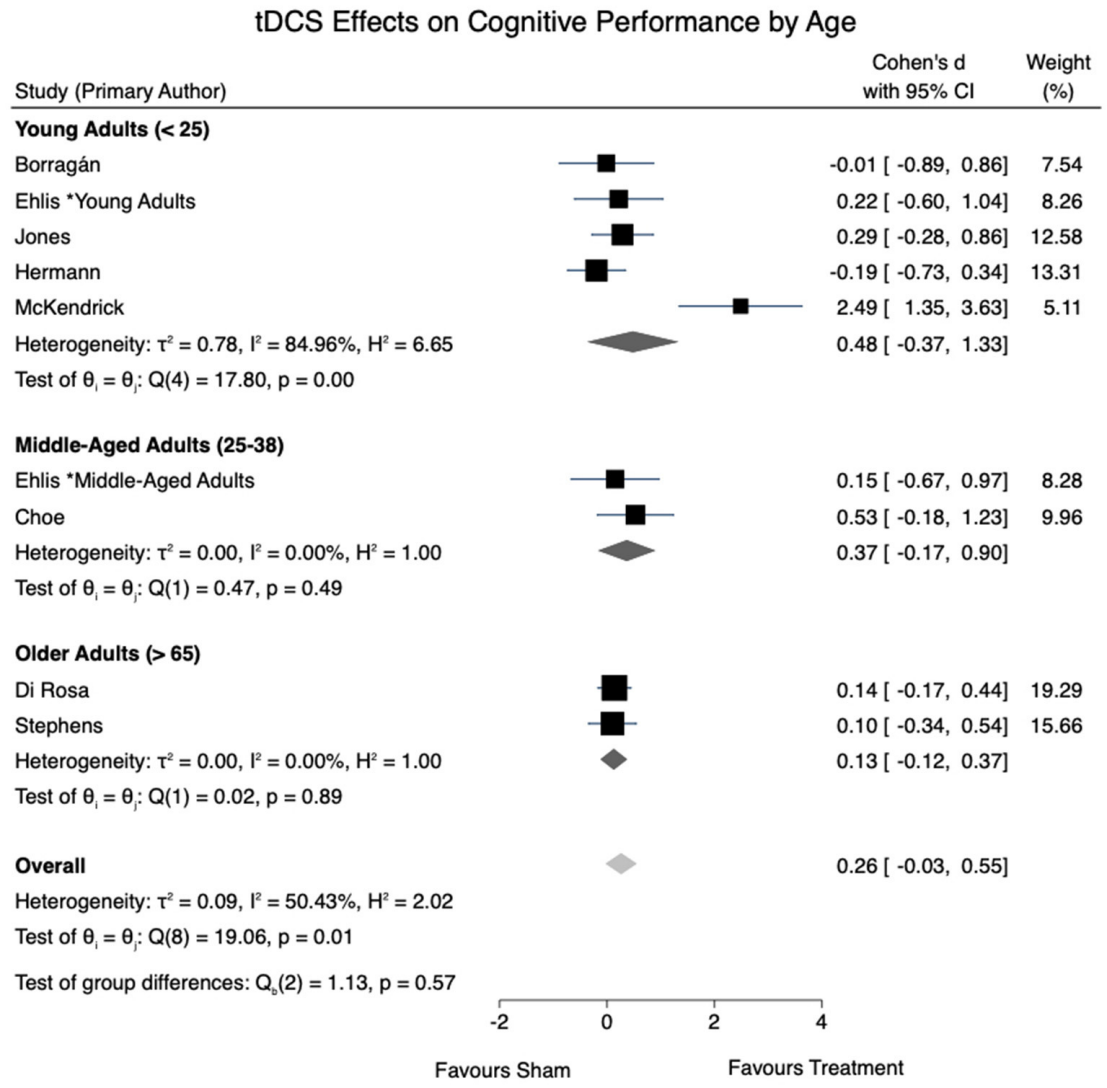
tDCS effects on cognitive performance by age.

All eight studies were eligible to be included in the cognitive performance meta-analysis. A moderate level of overall heterogeneity was observed [*I*^2^ = 50.43%, $\chi_{\left(8\right)}^{2}$ = 19.06, *p* =0.01]. An overall effect size for tDCS effects on cognitive performance of *d* = 0.26 (95% CI: −0.03 to 0.55, *p* = 0.077) was obtained. A non-significant trend-wise decrease in the effects of tDCS on cognition was seen in the pooled effect sizes of tDCS as age increased. Figure 2 provides a summary of the calculated tDCS effect sizes on cognitive performance.

### Impact of tDCS on fNIRS Outcomes

Studies differed in reported fNIRS measures (HbO, HbR, HbT, and calculated oxygenation metrics). Within the context of a cognitive task, three studies reported no effects of anodal tDCS on HbO (Choe et al., 2016; Stephens and Berryhill, 2016; Herrmann et al., 2017). Three studies reported an increase in HbO signals following anodal tDCS (Jones et al., 2015; Ehlis et al., 2016; Di Rosa et al., 2019), one study reported a trend-wise decrease in HbO signals following cathodal stimulation (Ehlis et al., 2016), and another study reported no cathodal tDCS effects (Herrmann et al., 2017). When considering HbR, one study reported an increase in HbR concentration within the frontotemporal cortex following anodal stimulation (Herrmann et al., 2017). Hemispheric differences were reported in two studies (Borragán et al., 2018; Di Rosa et al., 2019). Lastly, when examining oxygenation-derived values from HbO and HbR signals, two articles report decreases in regional oxygenation hemodynamic responses with anodal stimulation compared to sham (Borragán et al., 2018; McKendrick et al., 2020), fNIRS parameters are highlighted in Table 5 below.

**Table 5:** fNIRS parameters.

**Reference**	**fNIRS optode placement**	**Concurrent /sequential to tDCS**	**Signals reported**	**Recording parameters**	**Signal processing and analysis**	**Cognitive task measured with fNIRS**
Borragán et al., 2018	Bilateral Superior Frontal Cortex	Concurrent	COE (HbR-HbO)	Channels: 24 channels SDD: 3 cm Λ: 685 and 830 nm Sampling rate: 20 Hz Other: Triggered to event onset/offset of TloadDback task	Software: HomER Filter: Low pass (0.009–0.08 Hz) Analysis: Grand averaging of COE by 4 min blocks, ANOVA	TLoadDBack
Di Rosa et al., 2019	Inferior and Midfrontal Gyri, Supplementary motor area, intraparietal sulcus	Concurrent	HbO, HbR	Channels: 4 laser diodes and 8 photo-multiplier tubes. 38 channels, 2 short channels Λ: 690 nm and 83 nm SDD: 3 cm, Short channels: 0.8 cm Sampling rate: 7.8 Hz	Software: HomER2 Filter: Band pass filter (0.01 and 3 Hz); Corrections: Removal of signal-noise ratio <2 and motion artifacts. Age-dependent DPF. Consolidation: GLM approach of hemodynamic modeling with Gaussian functions. Mean HbO, mean HbR, mean hemodynamic responses in interval 5–11 s after stimulus onset. Analysis: ROI, ANOVA	Visuospatial WM task, reward incentives
Ehlis et al., 2016	Bilateral frontotemporal regions	Sequential	HbO, HbR	Channels: 44 channels (2 × 22) in two 3 × 5 optode arrays. Λ: 695 ± 20 nm and 830 ± 20 nm Sampling rate: 10 Hz	Software: MATLAB Filter: Low pass (0.3 Hz) Corrections: Linear fit function (10 s baseline, last 10 s of rest), noise correction by interpolation of mean adjacent channel signals Analysis: Means of the last 20 s of individual averaged activation was calculated (across each individual, condition, tDCS stimulation session, and channel). Channel wise t-maps, ROI Analysis, ANOVA	Verbal Fluency Test
Jones et al., 2015	Left prefrontal cortex	Sequential	HbO	Channels: 3 channels Λ: 690 and 830 nm SDD: 2.6 cm Sampling rate: 50 Hz	Software: HomeER2 Filter: Low pass filter (0.5 Hz) Corrections: Removal of first 5 s of each 25 s block and motion artifacts. Consolidation: Mean HbO per condition; recorded over final 20 s of each 25 s block. Normalization of HbO difference scores. Analysis: ANOVA	WM Change Detection Task
Herrmann et al., 2017	Bilateral prefrontal cortices	Concurrent	HbO, HbR	Channels: 52; Three rows (each with 11 optodes, SSD 3 cm). 33 optodes (17 laser diodes and 16 photodetectors) SDD: 3 cm Sampling rate: 10 Hz	Software: MATLAB Filter: Low pass (0.5 Hz) and discrete cosine filters Corrections: Removal of high-frequency artifacts using 5 s moving average, a common average reference to removing physiological noise, DPF. Analysis: Effect size (baseline to task performance), t-maps, ROI, ANOVA	Verbal Fluency Test
Stephens and Berryhill, 2016	Bilateral prefrontal cortices	Sequential	HbO	Channels: 14 Sampling rate: 50 Hz	Software: HomER2 Filter: Low pass filter (0.5 Hz); Corrections: Removal of motion artifacts. Normalization of each channel Analysis: Peak HbO amplitude per channel standardized per participant across time, transformed into an overall percentage of channels with decrease activation across time.	*n-*Back Task
Choe et al., 2016	M1, Right dorsolateral prefrontal cortex	Concurrent	HbO, HbR, HbT	Channels: 20 channels (10 channels over M1; 10 channels over the right dorsolateral prefrontal cortex). SDD: <3.5 cm Sampling rate: 8 Hz	Software: nirsLab, SPM Filter: Band-pass filter (0.01 Hz – 0.2 Hz) Corrections: Inter-trial signals removed from time-series. Average baseline concentration subtracted from task-evoked concentration changes Analysis: HbO, HbR, HbT average concentrations ran for each channel, participant, task, and time. Concentrations were averages within time (days) across all *n*-back trials. Concentrations were further region and grouped averaged across the total time difference. General linear model-based SPM was performed, multiple comparison correction of channels.	*n-*Back Task
McKendrick et al., 2020	Bilateral prefrontal cortices (Anterior and dorsolateral prefrontal cortices, Pars Triangularis, Pars Opercularis)	Concurrent	HbO, HbR, Oxygenation (HbO–HbR)	Channels: 16 Λ: 730 and 850 nm SDD: 2.5 cm Sampling rate: 2 Hz	Software: COBI Studio software Filter: Low pass filtered (0.1 Hz) Corrections: Motion artifact assessment Analysis: Temporal hemodynamic function temporally group averaged. Linear mixed effect modeling with restricted maximum likelihood. Bayesian information criterion to determine random and fixed effects. False discover rate corrections.	Spatial memory task

*COE, Cerebral Oxygen Exchange; HbO, Oxyhemoglobin; HbR, Deoxyhemoglobin; HbT, Total Hemoglobin; ROI, Region of Interest; ANOVA, Analysis of Variance; SDD, Source-Detector Distance; DPF, Differential Pathlength Factor; Λ, Wavelength*.

**Figure 3:**
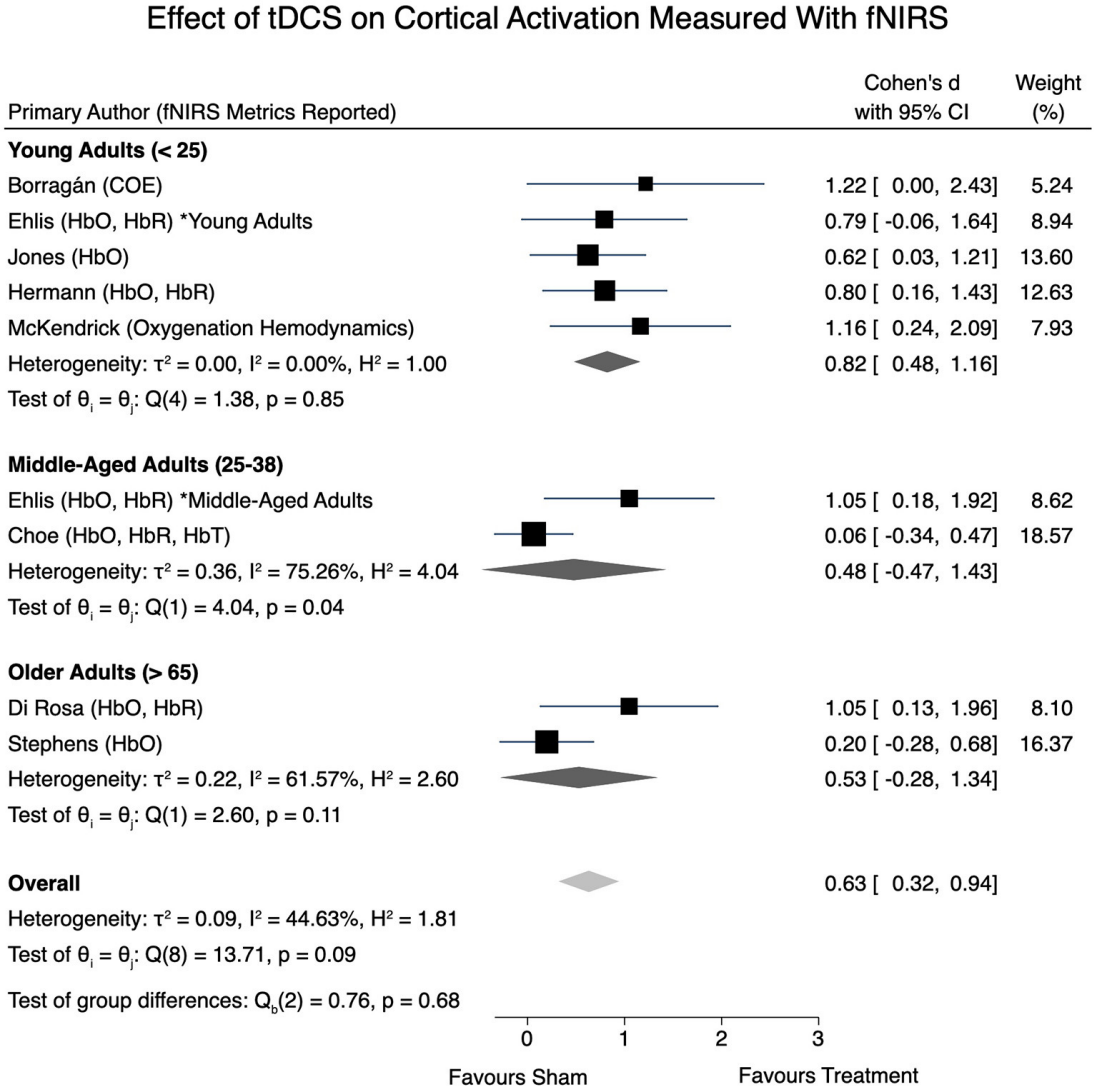
Effect of tDCS on cortical activation measured with fNIRS.

All eight articles were eligible for inclusion within the fNIRS meta-analysis. A moderate level of heterogeneity remained present when examining the overall effects of tDCS on obtained fNIRS signals [*I*^2^ = 44.63%, $\chi_{\left(8\right)}^{2}$ = 13.71, *p* = 0.09]. Effect sizes were calculated, however consideration of the signal directionality (i.e., if the effect size corresponds to an increase or decrease of an fNIRS signal) in the overall meta-analysis model was not taken into account. An overall effect size of *d* = 0.63 (95% CI: 0.32–0.94, *p* < 0.001) was obtained. Further, a statistically significant effect size of *d* = 0.82 (95% CI: 0.48–1.16, *p* < 0.001) was present in young adults, whereas non-significant effect sizes of 0.48 (95% CI: −0.47 to 1.43) and 0.53 (95% CI: −0.28 to 1.34) were determined in the middle-aged adult and older-aged adult groups, respectively. Figure 3 provides a forest plot of the included studies and their respective calculated effect sizes.

## Discussion

In this systematic review, we explored the effects of tDCS on cognitive performance and fNIRS-based hemodynamics. A secondary question explored how these measures are affected by aging. The studies reviewed included RCTs (*n* = 4) and within-subject crossover designs (*n* = 4). Four studies included young adults (mean age <25) (Jones et al., 2015; Herrmann et al., 2017; Borragán et al., 2018; McKendrick et al., 2020), two included older adults (mean age > 65 years old) (Stephens and Berryhill, 2016; Di Rosa et al., 2019), one included middle-aged adults (mean age between 25 and 38 years old) (Choe et al., 2016), and one study had both a young-adult and middle-adult group as participants (Ehlis et al., 2016). Based on the studies included in this review, tDCS does have an impact on cognitive performance and cerebral hemodynamics, as measured by fNIRS metrics. Further, as expected, aging processes appeared to alter the effectiveness of tDCS applications.

Five studies, all of which included young adults, reported no cognitive performance gains following anodal stimulation when compared to sham. Interestingly, in the subgroup meta-analysis, the pooled effect size was greatest in young adults under the age of 25 (*d* = 0.48), followed by middle-aged adults aged 25–38 (*d* = 0.37*)*, and older adults over 65 (*d* = 0.13). This trend was in the opposite direction from our initial hypothesis, which was based on previous reports of tDCS effects being greater in studies with older or cognitively impaired participants (Hsu et al., 2015; Summers et al., 2016; Ke et al., 2019; Nissim et al., 2019). Nonetheless, there are other reports of aging-related resistance to tDCS effects. For instance, Leach et al. (2019) reported tDCS-evoked cognitive gains in associative memory in young adults, which was absent in older adults in the same study. This is further in line with a previous tDCS meta-analysis specific to older adults, where no significant gains were reported in any cognitive domain (Horvath et al., 2015). Yet others have proposed that factors such as baseline performance or education level, as opposed to age, may modulate tDCS efficacy in older adults (Berryhill and Jones, 2012; Learmonth et al., 2015). Clearly, this is an area that warrants further study, and may even require tDCS protocols that are adapted to address the structural and neuroanatomical changes associated with aging brains (Habich et al., 2020).

For the purposes of the specific questions in this review, we included studies that explored the effect of tDCS on some aspect of cognition. Undoubtedly, there was much variability in the cognitive tasks used in the studies, including verbal fluency tasks (*n* = 2) (Ehlis et al., 2016; Herrmann et al., 2017), spatial memory tasks (*n* = 1) (McKendrick et al., 2020), and working memory tasks (*n* = 5) (Jones et al., 2015; Choe et al., 2016; Stephens and Berryhill, 2016; Borragán et al., 2018; Di Rosa et al., 2019). Within this latter category, there was a large amount of procedural variability. One WM task was a modification of the n-back task called T-load D-back, which incorporates both the n-back and a number decision task into one process (Borragán et al., 2018). Another was a novel visuospatial task that required both identification and location memory of pictures and letters (Di Rosa et al., 2019). A third study utilized an operation span task while another conducted a battery of n-back and letter span tasks (Jones et al., 2015). This heterogeneity in the behavioral assessment of WM introduces a potential reason/confound for the variability of tDCS effects. Though not within the scope of this review, two of the included studies further assessed the role of motivation on tDCS efficacy, both of which found that higher motivation via financial incentive augmented behavioral performance to a greater extent in anodal tDCS groups (Jones et al., 2015; Di Rosa et al., 2019). Further, one tDCS and fNIRS study examined additional variables related to flight simulation, however only the cognitive component was included in this review (Choe et al., 2016). It is possible the variation in effect sizes reported in this review is reflective of the differences in cognitive tasks used across the various studies.

The majority of articles reviewed utilized a working memory paradigm as the cognitive measure. The impact of tDCS on enhancing working memory task performance in younger adults has previously been reported (Katsoulaki et al., 2017). However, tDCS effect sizes within the cognitive domain of working memory also appear to differ across adulthood, and in older adults with mild cognitive impairment or dementia (Hsu et al., 2015; Mancuso et al., 2016; Stephens and Berryhill, 2016; Summers et al., 2016; Di Rosa et al., 2019). Although our search did not yield any studies of tDCS and fNIRS in individuals with cognitive impairments, this is a population in which further study could be illuminative of the impact of tDCS on cognitive performance and cerebral perfusion. Future investigations based on theoretical models of cognitive aging, such as the Hemispheric-Asymmetry Reduction in Older Adults (HAROLD) (Cabeza, 2002), Compensation-Related Utilization of Neural Circuits Hypothesis (CRUNCH) (Reuter-Lorenz and Cappell, 2008), and the Scaffolding Theory of Aging and Cognition (STAC) (Park and Reuter-Lorenz, 2009) may provide useful frameworks for further inquiry.

The studies reviewed lacked a standardized metric of cerebral oxygenation, reflected in the variety of fNIRS signals reported (Table 5). Even within studies reporting the same metric however, effects of tDCS on cerebral oxygenation were mixed. For instance, three studies reported increases in HbO following tDCS stimulation (Jones et al., 2015; Ehlis et al., 2016; Di Rosa et al., 2019) while three studies reported no significant changes (Choe et al., 2016; Stephens and Berryhill, 2016; Herrmann et al., 2017). One study reported an increase in HbR following anodal stimulation (Herrmann et al., 2017) and another two studies reported decreases in oxygenation when estimated as a function of HbO and HbR (Borragán et al., 2018; McKendrick et al., 2020). As there is little consensus on the downstream cognitive effects of changes in HbO and HbR concentration, this is an area where future studies may help to further elucidate the mechanisms underlying tDCS-induced cognitive enhancement.

In the studies reviewed, tDCS was found to impact cerebral perfusion as measured by fNIRS, demonstrated by our overall statistically significant moderate effect size of *d* = 0.63. We hypothesized that young adults would exhibit greater perfusional change relative to older adults following tDCS, as measured by fNIRS metrics. This hypothesis was supported by our subgroup analysis. A statistically significant effect size of *d* = 0.82 was present within the younger adults, whereas non-significant effect sizes were reported for middle-aged and older adults. It is possible that the large effect sizes calculated for the studies reporting decreased oxygenation (Borragán et al., 2018; McKendrick et al., 2020) may have skewed the overall effect size, therefore these results should be interpreted with caution due to the limited number of studies and level of heterogeneity present.

The theoretical grounding of this review is based on the premise that the interaction between the neuron (when modulated by tDCS) and associated cerebral perfusion at the neurovascular unit impacts cognitive performance. However, other changes beyond the level of the neurovascular unit, such as cerebral atrophy should be considered. In a study investigating cerebral blood flow changes across aging, Meltzer et al. (2000) noted there were no age-related cerebral perfusion differences using positron emission tomography (PET), after correcting for brain volume. This suggests that cerebral atrophy, not cerebral blood flow, may underlie functional deterioration, Conversely, another study using arterial spin labeling found that cerebral perfusion was significantly correlated with cortical thickness and total brain volume, as well as performance on executive function tasks (Alosco et al., 2013). However, there was no direct association between brain volume and cortical thickness with cognitive function. Another study using PET in participants with hypertension and lacunar infarcts or white matter lesions reported that lower cerebral blood flow precedes cognitive decline 3 years later, measured using the Mini-Mental State Examination tool (Kitagawa et al., 2009). From these findings, it appears possible that cerebral blood flow underlies a common mechanism present in both cognitive decline and cerebral atrophy.

No articles with individuals with MCI or dementia were identified in our search, demonstrating the need for cognitive-based tDCS and fNIRS research protocols with these populations. Significant effects of tDCS on cognitive performance have previously been reported in the literature (Cruz Gonzalez et al., 2018), and there is evidence the effectiveness of non-invasive brain stimulation may vary among older adults with MCI (Chu et al., 2021). Further research is needed to investigate potential age-related changes in cognitive mechanisms to explain this variability.

### Limitations and Future Directions

With the limited number of articles suitable for review, studies were grouped by age despite having varying cognitive tasks. Although spatial memory and working memory may represent similar cognitive mechanisms, verbal fluency tasks may be grounded in an alternative cognitive domain altogether. The studies using verbal fluency tasks were conducted in younger adults, which potentially impacted the effect sizes reported (Figure 2). Nonetheless, studies employing working memory tasks were included across all subgroups included in effect size calculations. With ongoing research in the field, it is recommended that future reviews conduct an analysis accounting for the different cognitive tasks utilized in addition to age.

Research investigating aging-related differences in tDCS and cognition as it relates to cerebral perfusion yields meaningful insight into the current understanding of these cognitive processes and the ability for neuromodulation. Future directions should also aim to investigate populations with microvascular changes (such as diabetes and chronic hypertension) in addition to larger vascular changes (such as aortic and carotid stenosis), using a cognitive-orientated tDCS and fNIRS paradigm to further assess the role of cerebral blood flow and vascular health in cognitive task performance.

There exists a possibility in which repeated tDCS sessions might induce different physiological changes within and beyond the stimulated brain region, and this should further be assessed within the context of cognitive aging. In addition to tDCS stimulation frequency, the effects of current intensity, time, regions of stimulation, and montage (anodal or cathodal) require further investigation regarding cognitive performance across normal and pathological cognitive aging. tDCS effects and direction of change (i.e., increases or decreases) of specific chromophores (HbO, HbR, HbT) or oxygenation is yet to be determined. With the limited number of cognitive-oriented tDCS and fNIRS studies, it is recommended that additional studies be conducted before establishing the directionality of these unknown variables in meta-analysis. With interindividual differences, it is recommended to perform electric field modeling using structural neuroimaging of each participant if available to assist in optimizing tDCS parameters and regions of stimulation. Lastly, to the author's knowledge, no widely available graphical user interface or software is available to model the effects of tDCS current on cerebral perfusion, which is an avenue to explore in the future using perfusional neuroimaging methods including fNIRS.

## Conclusion

With the eight included tDCS and fNIRS studies on cognition, we report significant overall effect sizes on cognitive performance and fNIRS signals due to tDCS-evoked neuromodulation. Further, age-related differences appear to alter the efficacy of tDCS effects. With the limited number of studies and heterogeneity in combined tDCS, fNIRS, and cognitive testing parameters, further research is required to test the efficacy and directionality of fNIRS signals. Confounding variables such as baseline performance, education, health status, and factors impacting cerebral blood flow should further be investigated and included in future study designs. In conclusion, tDCS may be a promising tool for neuromodulation and cerebral perfusion modulation, however, significant research is still needed to determine which groups are more susceptible to tDCS-evoked effects.

## Data Availability Statement

The original contributions presented in the study are included in the article/supplementary material, further inquiries can be directed to the corresponding author/s.

## Author Contributions

MF: conceptualization, methodology, investigation, validation, formal analysis, resources, data curation, writing–original draft, writing-review and editing, visualization, and project administration. MZ: conceptualization, methodology, validation, formal analysis, resources, data curation, writing–original draft, writing–review and editing, visualization, and project administration. EK: conceptualization, methodology, validation, formal analysis, resources, data curation, writing–original draft, writing–review and editing, visualization, project administration, and supervision. All authors contributed to the article and approved the submitted version.

## Conflict of Interest

The authors declare that the research was conducted in the absence of any commercial or financial relationships that could be construed as a potential conflict of interest.
